# Females and Their Diseases; a Series of Letters to His Class

**Published:** 1848-04

**Authors:** 


### REVIEWS.

---

ART. V.—*Females and their Diseases ; a Series of Letters to his Class.* By CHARLES D. MEIGS, M.D., Professor of Midwifery and the Diseases of Women and Children in the Jefferson Medical College at Philadelphia; Member of the American Medical Association; of the American Philosophical Society, and one of the Council; Fellow of the College of Physicians of Philadelphia; one of the Physicians to the Lying-in Department of the Pennsylvania Hospital, etc. Philadelphia: Lea and Blanchard. 8vo. pp. 670. 1848.

The first thing that will strike the reader of these letters is the free, off-hand, colloquial style in which they are written; and whether this will be felt to be a merit or a fault, will depend upon original taste, or the bias of education. The author himself had his doubts "whether such a mode of writing would prove agreeable to the brethren, so as to meet their approbation;" but he has ventured boldly upon the experiment, and if he has failed in it, he is not without hope that some one else may be more fortunate in finding a method by which we shall "get rid of our medical dullness." The style is certainly not faultless, but yet it is one which, we venture to believe, will prove acceptable to most of the readers to whom it is especially addressed. It is fresh, buoyant, varied and sprightly, and one is carried along by it without weariness, as by a lively conversation, which we deem to be no trifling merit in a work relating to such subjects. If the author had confined himself to our own copious language, of which he is so much the master, his brethren, we are disposed to think, would have been still better pleased with his style. Most of them will complain of his use of French words where English might have been better used, and some will object to his frequent quotations from the Latin poets. Since it was the design of the author to address the former members of his class in "the most familiar style," we wish he had confined himself to the use of the language with which alone they can claim to be familiar.

"As to the doctrine and the precept of these letters," we think with the author, that "he has a right, at his stage of life, to be heard upon them," and we are quite sure that he will be heard with great advantage. Whatever differences of opinion there may be respecting the manner of the letters, there can be no diversity as to the matter. They are full of instruction. It would be difficult to point to a volume containing more valuable information relative to females and their diseases. We shall attempt to make this appear by extracts from the work, and our first quotation is the entire letter on *Sex*. In this letter the reader will see the work of Professor Meigs. The volume contains no more characteristic letter than this, and few, perhaps, more interesting.

"You may remember that in lectures at the college I very frequently repeated that the ovary of the female gives to her the sexual character, and that as the interior and active tissue of the ovary is the part which Ch. Ernest von Baer calls the lager or stroma, so that very lager or stroma is the *sex* itself.

You might, perhaps, at first hearing this dogma, feel disposed to reject it as too concise an expression of the multitudinous and diversified characteristics of the sexual nature; but I hope a careful examination of the matter may induce you to coincide with me in opinion.

The sexual office is designed to reproduce the material forms and faculties of a genus or species; and this is not done save by the production of a germ, which, at least in all the zoological series, is found within an ovum, which in plain Englisn is an egg. *Omne vivum ex ovo* is a true saying.

An ovum, then, is an egg, which is not to be considered as a germ, but as a thing containing a germ and the material or pabulum for the early stages of the development of that germ.

An egg is a yelk-ball, which in some instances is invested with a quantity of albumen or white, and in some cases is not accompanied with any deposit of that sort.

The yelk-ball is a vitellus, and consists of a multitude of corpuscles and granules, and punctiform bodies and small globules of oil swimming in a clear, delicate fluid, inclosed by a delicate anhistous membrane, the vitellary membrane. In most cases, yelk is of a yellow hue, in some it is red or greenish, etc.

If you break open a yelk, whether of the humming-bird or the ostrich, of the elephant or the mare, the rabbit or the earthworm, the shad, the minnow, or the whale, you will find it to consist of corpuscles, granules and puncta, with oil globules and the clear fluid.

If the egg has not been subjected to fecundation, as it cannot be within the ovary, there will be found within it, at the center of the ball or near the surface of the vitellary membrane, a smaller spherule of a beautiful transparency, and which is denominated the germinal vesicle—within which, adhering to the wall of the vesicle, are a number of very minute microscopic granules, which by Rudolph Wagner, of Berlin, by whom they were discovered, are called the macula germinativa or germinal spot—so that an egg is, 1, a vitellus; 2, a germinal vesicle contained within a vitellus; 3, a germinal spot lodged upon the inner wall of the germinal vesicle.

Is it not to be believed that the germinal spot is the true germ-point or germ itself, and that all the other constituents of the ovum are placed round about it in order that by its changing and formative power, its metabolic and plastic force, it may convert their elements into its own corporeal elements, evolving by their consumption the rudiments of its own organs, as brain, veins, heart, blood, etc. etc., until, having acquired sufficient organic form and force to make its mesenteric attachment to the living parts of the womb, it is ready to be conceived—conception being the formation of that mesenteric attachment, and nothing more nor nothing less?

Nature—all whose operations are preordained as being guided and limited by the law of God—has provided fully and most liberally for the production of germs, and thereby secured the perpetuity of her kinds. She has, in order for this germ production, adopted various methods of arrangement. In certain animals she has devolved the germ-producing and the fecundating powers upon the same creature; in others she has provided two independent forms of being for the carrying on of the office.

In the lower orders of beings, as the earthworm, for example, both the germiferous and the fecundative attributes are comprised within the same individual body; so that the creature can fecundate its own female constitution by the act of its male constitution. And this simple but effectual mode of keeping up the genus or species is admirably adapted to the inactive and aperceptive nature of the being itself.

In the higher forms, two separate individuals are provided, one with the male or fecundating nature, and the other with the female or germinating nature. "Male and female created he them," unto the end that they might increase and multiply, and fill the earth with sentient beings wonderfully endowed with life-faculties, and therefore with the means of enjoyment, in other words, of happiness. To procure happiness and establish it must be regarded as one of the highest attributes of Divine power and beneficence.

The important, the indispensable ovum could not be left without protection; it could not be developed without a machinery.

The ovum is therefore protected with an ovisac or capsule, which is what is called a Graafian vesicle, or Graafian follicle, or Graafian cell, from Regnier de Graaf, a Dutch physician, who—in his 'Treatise de Mulierum Organis Generationi Inservientibus,' published in 1672—gave the first clear account of those small pellucid vesicles that you have so often seen in the ovaries I exhibited to you.

A Graafian vesicle consists of a double-coated capsule, of which the outer one lies in contact with the stroma, and contains the inner one within its own sphere—not loose and unattached, but connected to it by means probably of a very delicate laminated cellular tela.

This interposed and connecting cellular tela may become filled with secretions; and as the outer one is, like a mineral in its gangue, pressed against the stroma, it is clear that any interstitial deposit must have two contrary results, one to enlarge the outer concentric and throw it back against the stroma, displacing and distending it, and the other to press the ovisac inwards towards its contents, to compress them, and to render the inner aspect of the ovisac uneven, wrinkled, or convoluted; and, in fact, I have shown you on several occasions both these different results.

I pray you to call to mind not only the human ovaries in which I showed you the convoluted or wrinkled appearance of the inner aspect of the ovisac, but the more striking samples of the same effect in the ovary of the cow. In this latter case the interstitial deposit was so enormous that the outer coat of the ovisac had grown to a size nearly equal to half the mass of the whole ovary, and was of an orange hue, derived from the deposit of a vast quantity of vitellary matter betwixt the two laminæ; the outer one being greatly expanded or extended, and the inner one being crumpled, convoluted or wrinkled. This great orange colored mass was the corpus luteum.

Before the corpus luteum had begun to form, the ovary had been occupied in furnishing vitellary matter for the constitution of the yelk-ball; but that ball having become complete in all its parts, and incapable of any further accretion as contrary to its generic law, the ovarian stroma, whose office it is to produce germs and vitellus, could not at once withhold its vitelliferous power, and the deposit went on upon the outside of the ovisac.

Hence you perceive that in a perfectly mature Graafian follicle there is a yelk-ball with its germinal vesicle and germinal spot inside of it; some fluid of the ovisac filled with granules; while on the outer surface of the ovisac, between it and the external capsule or coat, is a yellow deposit, which begins to appear there about the time when the yelk is quite ripe, and continues to augment in quantity for some time after the yelk has been discharged. Let me repeat, that this yellow body is the famous corpus luteum, and that such a yellow mass is deposited with the maturation and discharge of every yelk. Sometimes, perhaps, in pregnancy, the corpus luteum is larger than at other times, but whether large or small, it is a real corpus luteum; for there are not, as some writers pretend, true and false, but only true corpora lutea.

But this gradual evolution of the luteal deposit has the effect of lifting the germ, with its vesicle and its yelk-ball, nearer and nearer to the superficies of the stroma, to press outwards the tunica albuginea, and finally to press it so forcibly that absorption commences at the weakest and most distended point of its surface, until, a pore being formed, the yelk and the fluid about it escape from their imprisonment in the ovary, and fall into the belly, or are received within the infundibular orifice of the oviduct, which you call the Fallopian tube, and thence conveyed to the womb. Its mesenteric attachment to the womb, as I before said, constitutes a conception, which pregnancy follows.

I have made the foregoing observations in order to fortify my assertion, that for the female, stroma is sex.

Do you think that if a creature should be born with the external genitalia perfectly well formed, with a perfect uterus, and vagina, and tubes, but without the trace of any ovarium; such a creature could be a female? Or if she should have two perfect ovaria, and be born without womb, or vagina, or external organs, would she be anything else than a female? I am sure you will agree with me, that she would not, in the former case, be female, and that in the latter she would be truly female—because, though unhappily deprived of any gestative organ or organ of copulation, she is endowed with the germiferous through her vitelliferous faculty, which resides essentially and exclusively in the substance called stroma. No germ could she evolve in the spleen or liver, in the kidney or brain, in the heart or lungs, nor, indeed, anywhere save in the stroma, which is the true sexual concrete, and is therefore itself sex; for liver, heart, brain, lung nor digestive canal is not sex, but stroma is sex for the female—nothing else is sex.

But, after all, what is sex? Methinks you ask the question, which is a very difficult one. Perhaps I should best answer it in a few words, by saying it is reproductive power, whether male power or female power. It is a matter of indifference whether the philolologists derive the word from *secus*, *aliter*, otherwise, after another manner, or from *secare*, to cut, to divide one from another. We have in physic less to do with philology than with facts. I consider that we are hitherto unacquainted with any facts that give convincing proof of a sexual nature in the germ.

There is an embryonal stage of life in which it is utterly impossible to determine the sex of the embryo; and it is not known whether the female embryo proceeds from a germ originally female, or whether the germ, being in its inchoate state neither male nor female, assumes the female nature in the progress of its evolution, or takes on the nature of the male under some law as yet unknown to us.

It is very certain, however, that for the human race the proportion of the sexes, as to their number on the globe, is maintained from age to age. The law that ordains this equable rate of production operates so as to bring into the world about 104 males to 100 females, a proportion which keeps the sexes nearly equal in number, it being supposed that the temperament and exposure of the male render him more liable to premature death than the female, on which account the excess of males is ordained.

If we refer to what is known to occur as to the nonsexual nature of the larva of the bee, hereafter to be mentioned, we may find arguments for the opinion that the germ originally is nonsexual, but becomes male or female under some unknown law of development in its earliest embryonal life.

If such a sentiment may be rightfully entertained, you will, perhaps agree with me, that the sex is something superimposed upon the mere living nature of a creature; and you will more readily admit of it if you contemplate two children, one male and the other female, of the same stature, weight and temperament, born at the same hour, and brought up at the same breast. You cannot report to the mother of what sex they be, without referring to the pelvic extremity of the trunk. They are pleased with the same rattle, tickled with the same straw; they play at the same toys, and are alike in moral and physical attributes until the sexual endowment comes to be granted to them. Upon that instant they divaricate; their whole physical and intellectual and moral forces become different, and they pursue, so to speak, a separate walk of life, until the exhaustion of the sexual attribute in the one and in the other causes their paths to converge again, until they are seen sleeping "*thegither at the foot*" of the hill of life, over which they had toiled in distinct tracks from the puberic until the critical age. What can be more like an old woman than an old man? or what can be more like a girl than a perfectly ingenuous boy? Where is the likeness of men and women?

Is it not true, then, that the sexual nature is something superadded to the mere living corporeal nature, which, on being taken away, reduces them back again to their original sameness of life-nature?

You have heard, I presume, of a circumstance that may tend to illustrate this view, in the history of the honey-bee. The community requires a queen—which means a vitelliferous and so a germiferous creature—and it also requires a considerable number of drones, males, or fecundating members of the society; which being provided for the hive in queston, nothing more is wanting but a sufficient number of mules, or working bees, or creatures capable, first, of enjoymet, and, second, able to provide for the conservation of the species by collecting food—1, for the germiferous; 2, for the fecundating, and, 3, for the providing part of the species. Now, all these males and workers are alike in the ovular state; but if the males die by some epidemic, a battle or accident, the community know how to convert the larvæ into males by administering to them certain sorts of food, or to leave them mere workers by withholding that kind of aliment. So that in fact you discover here that the mere corporeal life of the larva possesses no sexual nature, and that a sexual nature may be superadded by a certain economy of the hive—an economy that can cause the ovum so to develop itself as to become fitly provided with the fecundating apparatus and material, or to become a queen bee or germiferous bee; for when a queen dies, after having deposited many thousand eggs in the mule bee cell, the alarm and confusion in the hive are very extraordinary, but it subsides after the tumult of the first excitement, and the mules or workers select some one egg, for which they enlarge the cell by converting three common ones into a single royal cell; and then, by feeding the grub with an aliment called royal jelly, they cause it to pass into the female state; and thus the lost queen is succeeded by a queen produced from the egg of a mule or worker bee—an egg that could only have developed a nonsexual creature but for the special influences brought to bear upon it from a state of necessity. I ask you again, if this be true, whether it does not show that the sexual nature is not an original nature, but a nature superimposed upon a mere animal or living nature? And if true of the bee, does not that truth established likewise establish the law for all possible animal and even vegetable existences?

I see not how a better proof, or, at least, illustration, could be given of my idea, that the sexual nature is a climax; it is a culminating life-force that evolves it.

It ought not to excite our astonishment that the female sexual nature gives to her physical, intellectual and moral attributes a bias different from that of the male.

Her organs are different; they are subject to fluctuations as to the tide of life within them that those of the male are by no means exposed to. They require a different and more complex system of innervation, more expensive to the nerve centers than those of the male, more delicate, sensitive, impressible than his. These are circumstances implying a dependence and physical debility as compared with him—a reliance and trusting to his power—and, in fact, all the peculiarities that mark her as a creature of the feminine and gentle sex."

If it were our object merely to give an idea of the manner in which this work on a most interesting and important subject has been executed, we might close our notice with the foregoing extract. But such is not the fact. It embraces too much matter of moment to the practitioner, and too many physiological views of which no physician should be ignorant, to be dismissed with so slight a notice.

Having shown what, in the author's view, constitutes sex—a view in which, we suppose, all will concur—we pass over many intermediate letters, and proceed to notice some topics akin to this, discussed under the head of "*Menstrua*," For long centuries it was a matter of inquiry among the learned, what is the cause of menstruation in women, and the question has only just received a satisfactory answer. It is given in the following words:

"A healthy woman matures and deposits an ovum every twenty-eighth day, from the age of fifteen to that of forty-five years, failing only in case of pregnancy and lactation, and sometimes not even then. She sometimes suffers an arrest of the force during lactation, yet in the majority even that arrest is but of short duration, and in many it does not take place at all. The closing stage of the process of maturing and depositing or discharging the ovum is attended with a discharge of bloody fluid from the genitalia, which is called menstruation, because it takes place once a month."

As each blade of corn came from a grain and each oak from an acorn, so every animal, small as well as great, existed once as a germ. "The egg of the barndoor fowl is not more perfectly an egg than is the microscopic egglet you find in the Graafian follicle of a cow, a mare, a sheep, a dog, or a whale. Each egg contains not only its germ, but its yelk." Such is the received doctrine on this subject.

The germ is produced by the ovaria, behind the peritoneal coat, in the Graafian cells, matures, is discharged at the menstrual period, and, if not fecundated by the male, escapes and is lost. Our author remarks:

"At different times I have exhibited to you, and great numbers of the class have come down into the rotunda to look at it, the yelk taken from the ovary of the cow as well as that from the ewe. You remember that the ovarian vesicle was punctured with a lancet, and the drop of liquid which spirted from the incision being collected on a lamina of glass and placed under the microscope, the yelk containing its germinal vesicle and macula was shown you in one of Chevalier's microscopes. This yelk-ball was contained within Graaf's vesicle. Graaf's vesicle has two coats, an inner one, and an outer one which contains the inner one, sphere within sphere; but the spheres are buried beneath the fibrous coat or albuginea of the ovary. The albuginea is contained beneath the indusium or the peritoneal coat.

"This statement, taken in connection with the fact that the ova of birds, and fishes, and frogs, etc. are discharged without the intervention of the male or any antecedent sexual conflict, ought to convince any one that the fecundation of the ova does not take place within, but outside of the ovaria, and therefore the ova must escape from the ovaria previous to the impregnating act. In other words, there is a physiological function of the ovary to mature and discharge its ova, in order that they may be afterwards haply fecundated. If you admit that this statement is a correct one, then you accept the doctrine of the spontaneous expulsion of ova, or the *ovi-posit*. Admitting the ovi-posit as a law of the reproductive force, then the question arises again, is this an irregular or a regular and periodical function? I have shown that throughout all living nature it is periodical, not continual, not irregular, not accidental. I see no bar to the conclusion that it is so in women.

"M. Negrier, of Angers, M. Gendrin, Dr. Robert Lee, M. Bischoff, and, more than everybody else, Dr. Pouchet, of Rouen, have taken pains to observe the state of the ovaria in women who have died during or soon after the act of menstruation. They have all found that at that crisis the ovary exhibits a mark on its surface, a bloody spot, a hole, a pore—into which can be pressed the point of a probe. This hole, this pore leads to an expanded crypt filled with a minute clot of blood. If the ovary be split with a scalpel, conducting the incision so as to cut through the pore, it will be found to lay open a Graafian follicle, from which the egg has escaped through its pore-like hila, as happens in the ovary of the bird. These appearances are seen whenever an ovary is examined during or shortly after a menstruation. You may remember that I showed you such a one in the ovary of a young woman who died shortly after her menstrua, and, what is more, I showed you the trace or cicatrix of the menses that preceded the last one, and I laid it open and showed you its crypt not yet closed up within, though healed on the surface; and a specimen is now in the museum, which contains the uterus of a young person who died suddenly while the menstrua were present: the uterus still contained the fluid, and the pore was bloody. My own opinion is, that you will never examine the internal genitalia under such circumstances without finding the fresh spot, and that you will not find it under any other circumstances. All this has been so clearly made out by M. Negrier, and particularly by M. Pouchet, that it is a loss of time to talk about it by way of proof or corroboration; and I really suppose that he who does not see and admit the doctrine of a spontaneous ovi-posit coincident with menstruation, must close his eyes to the light, and deny the most irrefragable demonstration."

This being settled, there is no difficulty in accounting for the menstrual engorgement of the reproductive organs. Pouchet found that the sow, during menstruation, exhibits in the vagina a rose tint, and that the womb was highly red and injected. This condition of the uterus in the human female is relieved by the catamenial hemorrhage.

"I conceive that enough has been said to convince you that the ovulation and spontaneous deposit of ova is completely independent of the sexual congress; and you ought to add, completely independent of and disconnected with any sexual sense or sentiment in the human being, though it is far otherwise in the lower mammals, etc. The reason of this difference is to be found in the high morals of reasoning creatures, as distinct from those beings that are governed by instinctive sense, and not by reason.

"If you accept the doctrine of the spontaneous periodical deposition of ova, then I think you have little difficulty to account for the mensual engorgement of the reproductive organs, or the monthly local plethora, or turgescence, or hyperæmia, which is relieved so regularly and completely by the catamenial or menstrual hemorrhage.

"The evolution of a Graafian cell is more and more rapidly effected, as it approaches nearer and nearer to its completion. The largest and most mature follicle is now enveloped, so to speak, in a mass of injected and engorged and hyperæmic tissues redolent with life; it is surrounded with *red vessels* carrying on in it the development offices. The cell, like a growing tooth, is magnified and raised up so rapidly, that, like the gum over the tooth, the stroma around the cell becomes turgid, succulent, almost inflamed, we might say. Under such circnmstances, what wonder have you to find the whole ovary swollen and turgid, or the womb itself affected in the same way? What wonder to find the woman complaining of pain in the ovaric region and in the womb, with aching, with heat, with sense of weight and dragging in the pelvis; and to find, on examination, that the uterus is larger, heavier, and more colored than in the intermenstrual periods?

"In fact, the womb, previous to the disengorging outflow of the menstrual blood, is redder and heavier and more succulent than when it has been fully acquitted by the discharge."

The author advances a step further on this curious subject, and presents a theory, not yet admitted, of the manner in which the corpus luteum is developed. He says:

"The function of stroma is to produce germs and vitellus. When the production of the germ on the inner face of the vesicle buried within the vitellus is complete, the ovulum is ripe; it has attained its generical magnitude and weight; but the stroma continues to supply the vitellary matter; it deposits it betwixt the two concentric spherules of the follicle. The inner spherule yields to the pressure of the increasing mass, and becomes convoluted as it is crushed inwards towards the yelk-ball. The outer spherule is driven outwards against the gangue of the ovarian stroma, and the deposit goes on, particularly in the cow and the swine, to a vast extent, making, in some instances, a mass of corpus luteum equal to half the volume of the ovary.

"At the same time the inner concentric, growing smaller and smaller from the pressure towards its center, lifts the yelk ball to the albuginea and indusium, where the resistance is weakest. The cavity of the follicle becomes reduced nearly to nothing as the pressure augments, until at last, the porule being established by the absorbents or by rupture, the yelk-ball escapes, along with its granular membrane, in fragments, leaving a crypt, which becomes filled with coagulated blood, the crypt being contained within a yellow mass of vitellus called the corpus luteum, which, at some stage as yet unascertained, begins to be absorbed as the vitelliferous forces of the stroma become determined towards the development of another follicle. It would be unreasonable to suppose that upon the escape of the ovulum, the yelk-producing force and activity should be immediately suspended. It is even probable that the corpus luteum may continue to augment for a certain but unknown short period."

It results, therefore, according to this view, that the act of menstruation "consists in the periodical maturation and deposit of an ovulum," of which the discharge is but the outward sign. Hence a woman may menstruate regularly, without any hemorrhagic sign of menstruation. Our author remarks:

"Doubtless many women are perfectly regular who give no outward sign of it. Do you doubt the truth of this proposition? If you do, then I ask you if it be not proved by the fact that a great many women who give suck do not have their courses until they wean the child? It is true that a great many women during their lactation find themselves to become regularly unwell from the seventh month; and it is also usual to observe that a woman lying-in has a return of the courses six weeks after the birth of the child. This is so generally the case that I always expect my patient to be unwell again at the sixth week, and to be regularly menstruous from the seventh month. This is the rule; the exceptions are in those women who never *see* until they wean the child, of which class the number is large."

On the same point he has the following instructive observations:

"You ought further to learn, that some women do not *see* from the time of their first conception until they have borne a considerable number of children, because, becoming fecundated while they are nursing, they carry out the pregnancy to term, become again enceinte, and so on, never seeing their courses, as I said, until, being no longer impregnated, the ovulation is marked by a natural return of the mensual hemorrhage. I have met with several samples of this kind, and they are so abundantly recorded in our books that there is no need to cite the cases; you may set it down as a fact that it is so with not a few women. How could it be otherwise with those women who have a child every year or eleven months? I have seen a child born at ten months after its antecedent.

"Now, supposing the ovulation and spontaneous deposit of the ovule to be the true doctrine, do you not see that these suckling women in question did really produce ovules for fecundation, though they did not have the menstrual discharge? I deem it no matter of surprise that they had not the menstrual discharge, because I can perceive in the function of lactation, an action derivative from the internal genitalia to the mammary glands, of strength sufficient to turn aside the determinations, whether sanguine or nervous, of the constitution from the genitalia to the lactiferous apparatus. Many other things turn it aside in the same way. There are many women who may be presumed to have the inward menstruation, videlicet, the ovulation, without any bleeding—a case that may take place without the least shock to the woman's health. A young woman, for example, may have reached the proper age for menstruation without having seen the sign or show. She may be married, conceive, and bear a child, suckle it, and at the end of seven months have her first menstruation. This has been observed to happen more than once. Who can doubt that she matured and deposited her germs, which were fecundated after her marriage? Or will you go back to the idols, and embrace the old doctrine that the germs were fecundated in the ovaria? If you will go back to such idols, I have nothing to do but 'let you alone.'

"You might well suppose that pregnancy will put a stop to the ovulation; and perhaps in a majority of cases it is true that when the womb has once fairly begun its career of gravid development, the sanguine and nervous forces of the reproductive organs are so completely absorbed in the business of gestation, that the ovarian function is suspended, or proceeds but slowly and feebly at most. Yet there are pregnant women not a few who have regular mensual returns for three or even four months. I had a patient here who was regularly unwell up to the eighth month, when I attended her. She went out her time, and was laid at the ninth month.

"Moreover, some women have the most insuperable tendency to miscarry, and those miscarriages are found to take place at the period of their usual menstruation; in fact, the major part of the abortions and threats of abortion you are hereafter to meet with coincide with the woman's catamenial period—a fact very important for you to know, because you ought to take measures to obviate the danger at the time, and, when the time is passed, relieve the patient from the onus of an unnecessary treatment.

"All the foregoing may suffice to show you that you are not to take in hand every female who does not have the show just at the time it is expected, and that you will be at liberty to suppose that her constitution does not suffer the least injury; because, while she does not seem to be regular, she is in fact perfectly regular—that is to say, she regularly matures and deposits her germs, for that is the physiological act of menstruation, and nothing else is. This is what her constitution requires her to do. If she bleeds, it is well; but she is often found to be well even if she does not bleed a drop. Is it not so with the suckling woman? I am glad to have an opportunity to tell you these things, for I hope they may have the effect to make you keep clear sometimes of the drugging process which is too apt to be set on foot the moment a lady complains of amenorrhœa.

"In a former letter I said I had often seen young women lose their courses when brought to town and set on the school form, yet retaining all the outward appearances of valid health. Whenever I have been consulted as to such a one, I have made a very careful exploration of the rate and degree of the various functions; and when I have found that the intellection, the respiration, circulation, innervation, calorification, digestion, secretions, etc. are all normal, and that nothing is wanting save the menstruous show, I have let the patient alone, merely directing a close surveillance of her health, and proposing to interfere only in case some further signs of disorder should present themselves. The deviation in such cases I have attributed to the consumption of the nerve force, the *neurosity*, by the hemispheres, in the too constant operation of the powers of the intelligence. A holiday or a return to their homes cures them better than drugs. Do not, I pray, let me mislead you here. On the contrary, I repeat that the state of the whole constitution should be carefully explored, and when therapeutical treatment is evidently demanded, see to it that the true indications be fulfilled."

The ovum is matured and discharged quite independently of sexual congress. When it has been fecundated by the contact with the male sexual element, it begins to be developed, provided that a connection is established between it and the living surface of the mother. It is not until this "mesenteric attachment" takes place that the woman can be said to have conceived.

"Doubtless thousands and millions of germs become fecundated, but which never form mesenteric attachments, and are consequently lost; the womb is only pregnant when the mesenteric attachment is made. No matter where this mesenteric attachment is, the woman is pregnant when it is made. It may be effected in some part of the tractus of the Fallopian tube, or it may be made upon the surface of the ovary, the ovary being covered at the time by the fimbria of the tube. If the porule of the Graafian cell have been formed, and the male sexual element have been translated through the channel of the tube, and come in contact with the exposed ovule, still contained within the Graafian crypt, the ovule may be there fecundated, and may form its mesenteric attachment within the crypt, and then you will have an ovarian pregnancy, and there is not another way in which you can have one. It is probable that the germ may be fecundated within the grasp of the fimbria, and being thus endowed with the development power, it may fall into the peritoneal sack, and there form its mesenteric attachment, thus constituting a ventral pregnancy. An ovule, on its passage from the fimbria to the uterus, may become fecundated; it may be arrested in the narrowest part of the tube where it passes through the thickness of the uterus; the delay and the pressure are probably the causes why it sometimes forms its mesenteric attachment there, and develops itself in the substance of the uterus outside of its cavity, thus constituting what is called interstitial pregnancy.

"Conception is never natural, never right, never safe, except when it takes place in the cavity of the womb. Please not understand me as stating that fecundation always takes place out of the uterine cavity; it is probable that it most frequently takes place outside of the cavity, but it may take place within the cavity of the womb. The fecundation takes place in whatever place the contact of the sexual element happens to be made."

It is, therefore, only when the germ is exposed in the Graafian cell, or in its descent towards the vagina, that conception can take place. But it is a question not yet determined, how long the germ may be retained with susceptibility to fecundation. Dewees reports the case of a woman whose pregnancy, there was good reason to believe, dated three weeks after the disappearance of her menses. Physiologists generally hold that the germ remains susceptible for eight days, and the truth of the opinion is established by a single historical fact alluded to by our author, that "the Jewish women do not return to the husband's bed until eight days after the disparition, and yet they conceive and bear children." Prof. Meigs believes that the ovulum may preserve its vitality as late as the twelfth or thirteenth day, but that fecundation is most apt to take place the first day after the disappearance of the menses, and that the chances diminish every day thereafter. But we will leave these questions for others of a more practical character.

The method adopted by Prof. Meigs is to describe the various organs peculiar to the female, and in connection with them the diseases to which they are liable. His remarks upon most of these are brief—so brief, indeed, that the reader will not always find as full information as he may desire; but they embrace the leading points on the various topics, and his directions are laid down in terms likely to be remembered. It is a task in which we should be glad to engage, if our limits would permit, to follow him through his lively letters, and give a synopsis of the matter of each one; but as it is, we can only select here and there a few passages, referring the reader for more ample information to the work itself.

We suppose that displacements of the uterus are among the most frequent of female disorders, inflicting serious and lasting trouble upon their subjects. Concerning the most common of these our author has the following remarks:

"But how is a man to know that the womb is prolapsed, taking his information solely from the statements of the patient? He cannot discriminate between the many causes of the very same painful sensation—that painful sensation being the expression of distress arising from a great variety of states of the parts in the pelvis. The only way to ascertain very clearly what is the fault, is to examine by the touch, and even that is insufficient in a great many of the cases of complaint; I say insufficient, not to detect a prolapsion, but to disclose the whole nature of the malady.

"There is but one course to take when the patient declines to have her serious case inquired into, and that is to argue the point with her so as to convince her judgement and obtain her consent, or to decline assuming the responsibility of curing her altogether. It is surely better to have nothing to do with the conduct of a case of disease in which the absolutely needful information is withheld. A physician who acts without knowing why, is more mischievous often than a disease left to its own native tendencies. Now, to continue in the care of a case you cannot cure because you do not understand it, is no profit nor honor—the greatest profit being always attendant on him who makes the best and promptest cures, and the greatest honor too."

The author has undoubting faith in the efficacy of pessaries in this complaint. He says:

"The comfort derivable from this method of support is scarcely describable. A woman who for months had been unable to walk or even to stand without an ineffable sense of weakness and pain, moves freely and spontaneously after the adjustment, and, in short, is as greatly relieved as is the man who has his humerus reposited after a dislocation. To show you how great that relief is, let me tell you that I was called to a very pious citizen, who dislocated his humerus into the axilla, and who, when I arrived, was holding the right arm in an elevated position by means of the left hand, with which he supported it, as the least descent of the elbow gave him exquisite pain. I took hold of the limb, made the extension in the proper direction, and then, depressing the elbow, reposited the head of the bone in the glenoid cavity. As the orbicular head took its place, he ejaculated with the utmost unction, 'Bless the Lord, oh my soul! and all that is within me, bless and magnify his holy name!" He had occasion to ejaculate his thankfulness, for he passed from agony into perfect calm. It is true, I have never heard a female make such an ejaculation upon a repositio uteri, but I doubt not the relief has many times been almost as great in the one case as in the other."

Not a few practitioners entertain doubts respecting the efficacy of these instruments. Dr. Meigs holds the following confident language in regard to their powers in all cases not complicated by loss of parts:

"Those females, however, who labor under prolapsion from the mere descent of the vagina, arising from its relaxation or loss of tonicity, can be cured by the pessary. I take it for granted, that every living tissue has an inherent tendency to contract, and when that tendency is not carried out into execution, it is because something resists, antagonizes, prevents it from obeying its law. To support, then, a vagina at its normal elevation within the pelvis, is to take away the resisting, antagonizing, preventing cause, and to allow it with time to recover its normal density and solidity.

"I mean not to say that this is always the sole thing needful to the cure; but I do suppose that it is in many instances the only medical or chirurgical process demanded, since the freedom from pain or inconvenience that follows the timely application of a proper pessary sets the woman free from the bonds of her symptoms, and enables her to take advantage of the restorative power of diet, air, exercise, bathing, traveling, and other hygienic methods, the wise and prudent employment of which may be expected to repair the mischief of debility and relaxation, not as to the vagina alone, but as to every part and parcel of her living system.

"Where the woman's general health, however, is broken, and her great alimentary, respiratory and circulatory functions overthrown, we should gather by careful inquiry and observation the indications of treatment, and pursue them to a subduction of the special evil or evils. A woman may have bad health from deranged action of the chylopoietic organs, requiring the exhibition of blue pill, taraxacum, alkalies, nitromuriatic acid, eccoprotics, or even purgatives. She may have neuralgic or neuropathic affections, springing from a vice in the hæmatosic tissues, from exaggerated vital sensibility of the heart, etc. In all these varieties of complaints, whose name is legion, let the especial sin be found out and eradicated.

"The bitter tonics, bark, wine, the chalybeates, a trained health, the exact indication of the diet as to quality and quantity, the amount of wine, malt liquor, etc.—these are the problems you are to solve, and upon their correct solution depends the question whether you are to have the great satisfaction of seeing your patient restored to health; or whether she, by a dawdling, indeterminate course of counsel and prescription, is to be left to drag out the weary years of broken health, lapsing from one evil to another, until, under the first serious attack of disease, she falls the victim of what is truly denominated a broken constitution. Who broke that constitution? The disease by its violence, or the doctor by his want of foresight, zeal and intelligence?

"The pessary, then, is to be regarded as the suspensory, as the splint, as the bandage; and, in truth, as happens in many other cases of surgery, these are the only indications. But as in surgery you would not, perhaps, treat an orchitis solely by the suspensory, but would make certain prescriptions, with a view to abate constitutional or local derangements of the circulation, the absorption, or the innervations of a part, so in the management of the prolapsions, you might not rest content with the curative power of the pessary alone, but provide the other juvantia of which I have already made mention.

"How long shall a woman wear the pessary? An important question, that cannot be solved but upon oxperiment. Several months will in general be required in any case, because the fastidious delicacy of a female will always prevent her from disclosing to you her distress in its early stages, and you know that chronical disorders are more difficult of cure than recent or acute ones. Hence I repeat that the cases that may come under your care are very likely to prove tedious and protracted; they will be mostly chronical."

We take leave of these Letters with the conviction that they will be productive of great good. They will be read with attention by many who would not have patience to wade through an elaborate, systematic treatise on diseases of females, and there is something in the dashing, random style which serves to impress their sentiments upon the memory. We do not undertake to say that the style is the best; it would probably be hazardous to assert that it is even a proper one for such a subject; but we must say, that it has contributed its share towards the great pleasure with which we have read this volume. Certainly we should be very far from advising the pupils of the author to become his imitators in this respect, but a book, now and then, thrown off in this careless manner, the grave scientific matter interspersed with anecdote, humor, gossip, poetry and sentiment, gives variety to our professional literature, and is not amiss. It pleases the fancy, while it instructs the judgement and stores the memory, and affords the reader a hearty laugh when he is least expecting it.

